# Dr. M. H. Cryer’s Visit to Dublin, Ireland

**Published:** 1903-10

**Authors:** 


### Foreign Correspondence.

### DR. M. H. CRYER'S VISIT TO DUBLIN, IRELAND.

To the Editor:

Sir,—Dr. M. H. Cryer, Professor of Oral Surgery in the University of Pennsylvania, gave a most interesting lecture and demonstration of dental anatomy on Wednesday evening, July 15, 1903, in the Albert Hall of the Royal College of Surgeons in Ireland. A large and intelligent audience listened to Dr. M. H. Cryer and examined the numerous specimens and preparations that were placed on the table for the information of the physicians, surgeons, and dentists who had been invited to attend.

The meeting was arranged by the Irish Branch of the British Dental Association, by whom Dr. Cryer had been invited to visit and deliver his most interesting and original lecture. The president and the vice-president of the College were in attendance, as well as members of the Council and the chief specialists in eye, nose, throat, and ear diseases, which are so closely related to the area involved in the practice of dental surgery.

The following evening Dr. Cryer was invited by the Irish dentists to a complimentary dinner at the Bray Head Hotel, county Wicklow. The company consisted of the guests and members of the Association. Mr. Joseph Thompson was in the chair, in the unavoidable absence of the branch president, and he proposed the health and prosperity of Dr. M. H. Cryer in felicitous terms. Dr. Cryer's reply was full of interest and information on matters that have vexed the dental world on both sides of the Atlantic for years past. Dr. Cryer's lucid and seasonable speech was closely punctuated with applause and cheers as it overflowed with a cordial good will towards the ideals as well as the practical efforts of the members of the British dental profession to advance their skill and usefulness. Dr. Cryer's peroration expressed his gratification of the warmth and cordiality of his reception so racy of the soil.

Before leaving Dublin the members of the Association presented Mrs. Cryer with a gold brooch of interlined Celtic design carefully copied from one of the panels of the famous Cross of Cony. Dr. Cryer was presented with a handsome example of a "Tipperary rifle,"—*i.e.*, a choice blackthorn mounted in gold with a suitable inscription engraved in Celtic lettering, which was reinforced with two *svaticus*.

Dr. Cryer's visit has been an occasion of much gratification to the members of the dental profession throughout Ireland.

[EXPLANATORY REMARKS.—The foregoing interesting communication from W. Booth Pearsall, Dublin, was sent to an influential journal for publication; but that not having a department for personal matters, it was transferred to the INTERNATIONAL, with the request that it be published. This journal very willingly complies with the request of our contemporary, as the account of Dr. Cryer's visit to Ireland will, without doubt, be read with pleasure by his many friends in this country.—ED.]

